# Computer-based comparison of different methods for selecting mitral annuloplasty ring size

**DOI:** 10.1186/s13019-017-0571-y

**Published:** 2017-01-30

**Authors:** Sameer Al-Maisary, Sandy Engelhardt, Bastian Graser, Ivo Wolf, Matthias Karck, Raffaele De Simone

**Affiliations:** 1 Department of Cardiac Surgery, Heidelberg University Hospital, Im Neuenheimer Feld 110, 69120 Heidelberg, Germany; 2 Department of Computer-Assisted Medical Intervention, German Cancer Research Center (DKFZ), Heidelberg, Germany; 3 Department of Computer Science, Mannheim University of Applied Science, Mannheim, Germany

**Keywords:** Mitral valve, Annuloplasty ring, Sizing, Implant

## Abstract

**Background:**

Ring sizing for mitral valve annuloplasty is conventionally done intraoperatively using specific ‘sizer’ instruments, which are placed onto the valve tissue. This approach is barely reproducible since different sizing strategies have been established among surgeons. The goal of this study is to virtually apply different sizing methods on the basis of pre-repair echocardiography to find out basic differences between sizing strategies.

**Methods:**

In three-dimensional echocardiographs of 43 patients, the mitral annulus and the contour of the anterior mitral leaflet were segmented using MITK Mitralyzer software. Similarly, three-dimensional virtual models of Carpentier-Edwards Physio II annuloplasty rings and their corresponding sizers were interactively generated from computer tomography images. For each patient, the matching annuloplasty ring was selected repeatedly according to popular sizing strategies, such as the height of anterior mitral leaflet, the intercommissural distance and the surface area of anterior mitral leaflet. The areas of the selected rings were considered as the neo-surface area of the mitral annulus after implantation.

**Results:**

The sizing of the mitral valve according to the height of anterior mitral leaflet (mean ring size = 29.9 ± 3.90), intercommissural distance (mean ring size = 37.5 ± 1.92) or surface area of anterior mitral leaflet (mean ring size = 32.7 ± 3.3) led to significantly different measurements (*p* ≤ 0.01). In contrary to intercommissural distance, height and surface area of the anterior mitral leaflet exhibited significant variations between the patients (*p* ≤ 0.01). The sizing according to the height of anterior mitral leaflet led to the maximal reduction of the mitral annulus surface area followed by the sizing according to the surface area of anterior mitral leaflet and finally by the intercommissural distance.

**Conclusions:**

This novel comprehensive computer-based analysis reveals that the surveyed sizing methods led to the selection of significantly different annuloplasty rings and therefore underscore the ambiguity of routinely applied annuloplasty sizing strategies.

**Electronic supplementary material:**

The online version of this article (doi:10.1186/s13019-017-0571-y) contains supplementary material, which is available to authorized users.

## Background

Mitral valve repair is a common procedure in the field of cardiac surgery. Despite the huge experience in this field, there is no standard approach to repair the mitral valve. Most techniques were developed empirically including the implantation of annuloplasty rings in order to reshape, stabilize or downsize the valvular annulus such that a sufficient amount of leaflet tissue is involved in coaptation. As the usage of prosthetic implants of suboptimal size or repair without ring insertion has been shown to adversely affect surgical results [1, 2], choosing the adequate ring size can be seen as critical [3, 4].

**Fig. 1:**
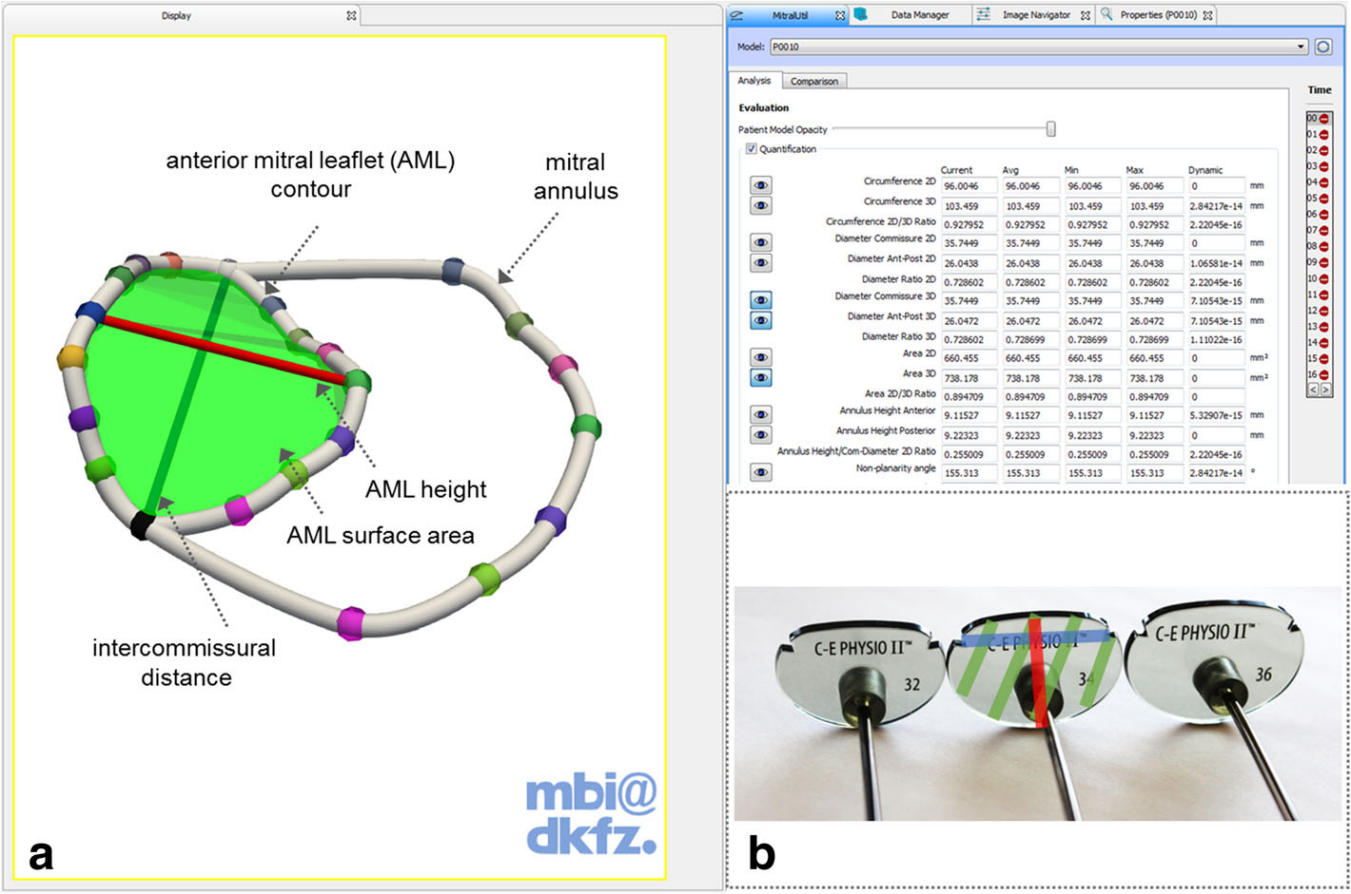
**a** Screenshot of the MITK Mitralyzer plugin with a modelled mitral annulus and anterior mitral leaflet. Furthermore, three anatomical measurements are shown, which are important for ring sizing. **b** Corresponding measurements on the sizer instrument

To date, one of the most commonly used prosthetic ring is the commercially available Carpentier-Edwards Physio II mitral ring (CE ring), which comes in 9 different sizes (size 28–40) [3]. Over the past decades, different strategies to find out the appropriate ring for various pathologies affecting the mitral valve apparatus have been developed. For choosing the suitable annuloplasty ring, template like translucent “sizer” instruments are used (see Fig. 1b), which are roughly similar in size like the prosthetic ring. They are placed onto the valve in-situ by the surgeon according to specific anatomical relations, such as the distance of the trigonal area, the intercommissural distance, the size of the annulus, the height or the surface area of the anterior mitral leaflet (AML). The AML has been preferably considered for sizing, since its tissue contributes mostly to mitral orifice occlusion upon systole; therefore the annulus should have approximately the size of the AML after ring implantation to allow the remaining AML tissue to coapt. Studies have proposed a vast amount of techniques to use the mentioned landmarks to find the best suitable ring according to the different pathologies and anatomical variations [3, 5–21]. To complicate the matter, concepts of “truesizing” and “downsizing” have emerged, which means that either the best matching ring is selected for the patient or one or two sizes smaller to reduce the size of the potentially dilated mitral orifice.

The advent of real-time three-dimensional transesophageal echocardiography (3D TEE) has allowed impressive volume renderings of the mitral valve anatomy and dynamics, especially the annulus, the leaflets and the papillary muscles, thereby significantly improving diagnostic capabilities and therapy planning. Additionally, the valve can be segmented from these images such that patient-specific virtual models are created. A specific software plugin called “Mitralyzer” of the freely available Medical Imaging Interaction Toolkit (MITK), which is able to process medical images [22, 23], was developed by our group for interactive and semi-automatic 3dimensional mitral annulus modeling [24]. The software allows for precise virtual reconstruction of the mitral annulus curve and the AML contour using 16 defined points on mitral annulus and the anterior mitral leaflet (see Fig. 1). The MITK plugin is planned to be released as a freely available software package soon.

Our goal was to study the effect of different sizing methods commonly used to implant a CE ring with regard to ring size selection, i.e. to find out if various strategies lead to the same results.

## Methods

Intraoperative real-time three-dimensional transesophageal echocardiographs of 43 patients, who underwent mitral valve surgery due to insufficiency, were extracted using Live-3D mode of the Philips ultrasound system iE33 xMatrix and a Philips X7-2 t matrix array transducer (Philips Healthcare, Andover, MA, USA). The images were taken intraoperatively on the beating heart and before initiation of the cardiopulmonary bypass. Only patients with good quality images that involve the whole mitral annulus and the mitral leaflets with the subvalvular apparatus were involved in this study. The images were transferred to a workstation running Philips QLab Software and exported in DICOM Cartesian file format to enable further processing. Using MITK Mitralyzer software with mitral valve images at early systolic phase of the cardiac cycle allowed the interactive generation of mitral annulus models and the contour models of the AML. Carpentier-Edwards Physio II annuloplasty ring sizers and Carpentier-Edwards Physio II annuloplasty rings of size 26–38 were digitized using computer tomography (CT, Somatom Denition, Siemens Medical Systems). The images were exported to DICOM format and a three-dimensional model representing each planar sizer (sizer model) and its corresponding annuloplasty ring (ring model) were made using MITK Mitralyzer. By the help of the software, automatic quantifications could be derived from the models, such as surface area of AML model, height of the AML model, intercommissural distance (same for both models) for each patient (see Fig. 1). From the sizer models the corresponding measurements (width, height, area) were computed automatically. Also the orifice area of each ring model was calculated using delaunay triangulation.

For each patient’s mitral annulus and AML model, we virtually selected the matching sizer model according to the following five different sizing methods: 1) corresponding height of AML (true sizing); 2) corresponding intercommissural distance (true sizing); 3) intercommissural distance with one size smaller (downsizing); 4) intercommissural distance with two sizes smaller (downsizing) and finally 5) corresponding AML surface area (true sizing). The anatomical references for the “true sizing” strategies are illustrated in Fig. 1.

Subsequently, the conformity between the results of the five strategies, i.e. the selected ring size, was compared to each other for each patient separately. The surface area of the chosen rings was considered as the surface area of the corresponding mitral annuli after annuloplasty.

The collected data was analyzed using analysis of variance (ANOVA) and *t*-test. Results are presented as mean ± standard deviation in mm for measurements and without standard unit for ring sizes. A *p*-value less than 0.05 was considered significant.

## Results

The mean age of the 43 patients at the time of the operation was 63.7 ± 11.8 years. About 77% of the patients were male. Thirty-four patients received mitral valve repair (81%) using Physio II annuloplasty ring. Eight patients (24%) of them needed an additional implantation of artificial polytetrafluoroethylene neochordae during mitral valve repair while sixteen patients (47%) received leaflet repair procedures and seven patients (21%) received both neochordae and leaflet repair. Nine patients underwent mitral valve replacement. More detailed characteristics of the patient cohort are summarized in Table 1.

**Table 1:** Patient characteristics

Preoperative data	
Age [years]	63.7
Female [number]	12
Coronary artery disease [number]	14
Atrial fibrillation [number]	8
Preoperative left ventricular ejection fraction (LVEF) [number]	
≥40%	39
30-39%	2
≤30%	2
Etiology of mitral insufficiency	
Degenerative [number]	39
Ischemic [number]	4

**Fig. 2:**
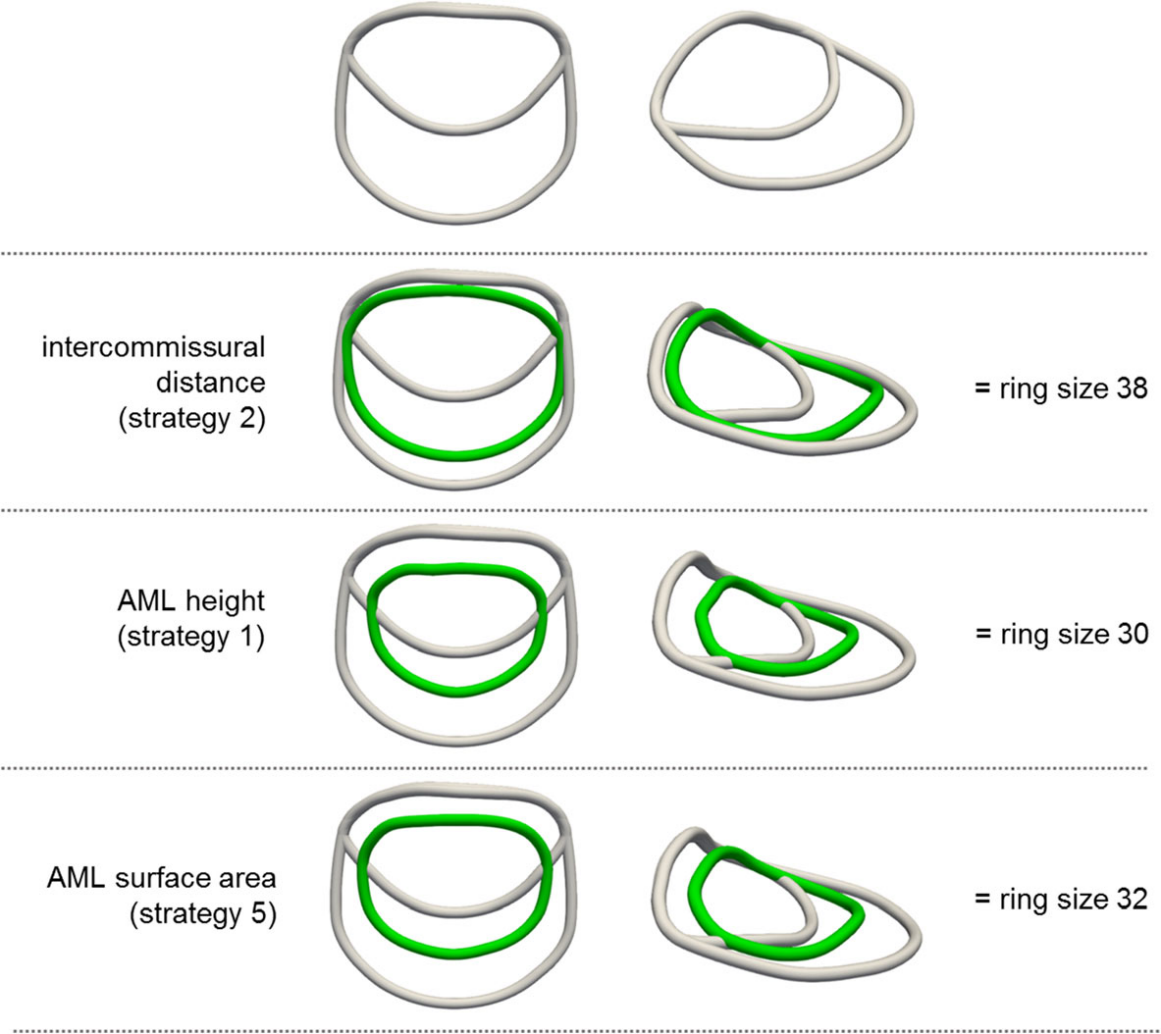
Averaged mitral annulus and AML model of all 43 patients (*white*) with the matching annuloplasty rings (*green*) according to the “true sizing” strategies. Mean ring size values were rounded to real commercially available sizes

Considering the whole patient collective, the sizing of the mitral valve led to significantly different average measurements (*p* ≤ 0.01) according to the five applied strategies: height of AML (mean = 29.9 ± 3.90), intercommissural distance with true sizing (mean = 37.5 ± 1.92), intercommissural distance with one size smaller (mean = 35.5 ± 1.65) or intercommissural distance with two sizes smaller (mean = 33.6 ± 1.38) and AML surface area sizing (mean = 32.7 ± 3.29). Fig. 2 illustrates this by showing the averaged mitral annulus and AML morphological model for the 43 patients together with the outcomes of the mean “true sizing” strategies (rounded to commercially available sizes).

Measurements of mitral valve apparatus landmarks are provided in Table 2. They showed significant variations in our patients’ cohort with exception of intercommissural distances. Height of AML measurements in our patients cohort (height AML mean =23.9 ± 3.74 mm) shows a significant variation between the patients (*p* ≤ 0.01). Intercommissural distance measurements in our patients cohort (mean = 38. 9 ± 6. 15 mm) shows no significant variation (*p* = 0. 31). The mean AML surface area was 711.13 ± 175.56 mm^2^ with significant variation between the patients (*p* ≤ 0. 01).

**Table 2:** Measurements of the studied patient cohort of 43 patients in systole

	Mitral anulus	Anterior mitral leaflet
Surface area [mm^2^]	1413.09 ± 338.31	711.13 ± 175.56
Height [mm]	38.0 ± 5.26	23.9 ± 3.74
Intercommissural distance [mm]	38.9 ± 6.15	

Mean and standard deviation are provided

The most sizing methods typically rely on measurements of the AML due to its tissue surface contribution to mitral valve closure, such that we did not consider the posterior mitral leaflet. Therefore, we compared the complete surface area of virtually implanted annuloplasty ring with the surface area of the AML. If the implanted ring was smaller than the AML area, we assumed that the implantation of such a ring will lead to a reduction of the visible AML surface area that participates in the mitral valve orifice during systole. The mean reduction in AML surface area when using height of AML for sizing was 116.7 ± 137.05 mm^2^ (16.4% reduction in AML surface area) while the reduction in AML when using AML surface area sizing was 19.43 ± 132.37 mm^2^ (1.2% reduction in AML surface area).

Using the intercommissural distance with true sizing (strategy 2), intercommissural distance with one size smaller (strategy 3), or intercommissural distance with two sizes smaller (strategy 4) led to no reduction in AML surface area as the implanted rings were 241.1 ± 164.86 mm^2^, 125.46 ± 163.06 mm^2^ and 30.24 ± 163.93 mm^2^ bigger than the AML surface area respectively.

Mean mitral annulus area was 1413.09 ± 338.31 mm^2^. The reduction in annulus area when using height of AML for sizing was 818.665 ± 296.3 mm^2^ (strategy 1) while the reduction in annulus area when using AML area sizing (strategy 5), intercommissural distance with true sizing (strategy 2), intercommissural distance with one size smaller (strategy 3) and intercommissural distance with two sizes smaller (strategy 4) were 721.39 ± 249.45 mm^2^, 460.818 ± 311.75 mm^2^, 576.493 ± 315.29 mm^2^ and 671.715 ± 318.64 mm^2^ respectively.

**Fig. 3:**
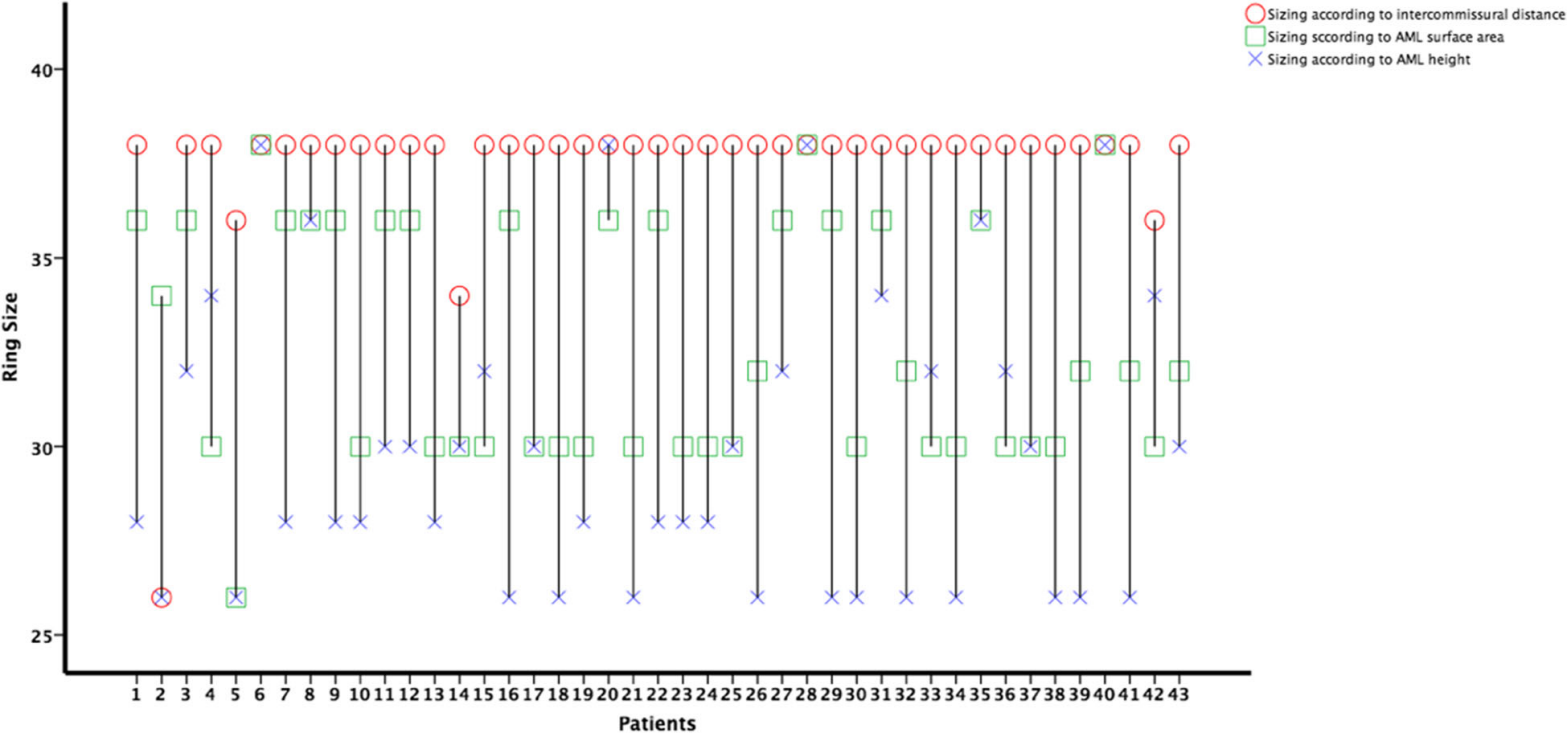
Sizing results for each patient according to the true sizing strategies: intercommissural distance, AML height, and AML surface area

The sizing results according to intercommissural distance (strategy 2) and AML surface area (strategy 5) led to the same sizing results in 14 patients (33%). For AML height (strategy 1) und AML surface area (strategy 5), ring sizing was equal in 10 patients (23%). In 4 patients (9%), the sizing results were equal according to AML height (strategy 1) and intercommissural distance (strategy 2). Note that in only 2 of the 43 patients (5%), the ring sizing in all methods was equal (see Fig. 3).

The actually used intraoperative method to size the mitral valve as conducted by surgeons are not documented. However, the mean size of the used mitral annuloplasty rings was 32.11 ± 2.44 and no obvious relation was found between the implanted rings and the virtually proposed rings. From our results, the outcome of AML surface area sizing method (mean = 32.7 ± 3.29) has its closest relationships to the actual implanted ring size.

## Discussion

Mitral valve reconstruction remains one of the most challenging fields in cardiac surgery with continuously changing concepts and new emerging ideas. The purpose of the current computer-based study was to find out if some of the currently used intraoperative methods for sizing the mitral valve annulus, as part of mitral valve repair using the CE physio II annuloplasty ring, result in similar ring size choices. The use of echocardiographic measurements to figure out the suitable annuloplasty ring is gaining more importance especially if three-dimensional measurements are performed [25]. The mostly used dimensions are the height of the AML and the intercommissural distance. Furthermore, the effect of different annuloplasty rings on the mitral valve geometry has been studied in-silico [26, 27] and in animal models employing radiopaque markers [28].

In our study, the use of the height of the AML (strategy 1) as reference to implant the annuloplasty ring was associated with the highest reduction in mitral valve orifice area and reduces the visible AML surface occupation area of the orifice; this theoretically implies the highest increase in coaptation area between AML and posterior mitral leaflet (PML). Such an increase in the coaptation area may result in a better competence of the mitral valve but may also lead to displacement of the AML causing systolic anterior movement (SAM) and left ventricular outflow tract obstruction with mitral insufficiency especially in the presence of a prominent PML. It may also cause iatrogenic mitral stenosis due to abundant valve tissue in the mitral valve orifice. Such postulations need further studies and finite element-based patient-specific annuloplasty simulations methods might be viable approach for this, such as shown by Schoch et al. [26]. However, it is crucial to take the height of the into account to avoid such complications. Many surgeon use AML height to better assess the dimensions of the mitral valve dimensions, but they also take the intercommissural distance in consideration to avoid excessive downsizing [3, 5, 14]. Note that in our study we only considered isolated strategies so far to show their single unaffected outcome.

Using the AML surface area (strategy 5) as a reference for sizing respects the AML and leads to less reduction in the mitral valve opening area which seems to be the best method to preserve the AML function but at the same time compromises the size of coaptation area [6], which considered as measure for the valves’ functional integrity. However, the accurate measurement of the AML surface area is more complex than the other chosen parameters, since we only contoured the AML edge but did not model the exact leaflet curvature. When comparing the outcome of AML height and intercommissural distance sizing, AML surface area sizing provides intermediate results for which most of the surgeon are somehow adapted to according to their experience [10, 11, 13].

Our study showed that sizing according to intercommissural distance results in the implantation of bigger annuloplasty rings (see Fig. 3), even if downsizing is used, which would in turn less decrease the opening area of the mitral valve in comparison to the other presented strategies. This implies that the PML need to play a more crucial role in mitral valve competence. Therefore, many surgeon use this dimension as the main sizing strategy while others combine the intercommissural distance with other dimensions to find out the optimal annuloplasty ring for their patients [3, 5, 20].

To further complicate sizing, it has been already shown by Bothe et al. [4], who termed the application of sizer instruments a “voodoo”, that there is a size discrepancy between corresponding rings and sizers which limits the scientific value of this procedure. Our comparison presented in Table 3 after CT imaging and interactive modelling of rings and sizes are in concordance with the findings by Bothe et al. [4].

**Table 3:** Comparison of measurements from CT-scanned and modelled Physio II annuloplasty ring-sizers and Physio II annuloplasty rings

Size	Sizer			Ring			Differences between ring and sizer		
	height [mm]	width [mm]	area [mm^2^]	height [mm]	width [mm]	area [mm^2^]	height difference [mm]	width difference [mm]	area difference [mm^2^]
26	21.12	26.26	478.37	19.37	26.20	424.45	1.75	0.07	53.92
28	22.91	27.97	434.61	21.10	28.13	541.36	1.81	−0.16	−106.76
30	24.41	27.11	583.21	22.26	29.99	559.00	2.14	−2.87	24.21
32	24.95	27.55	561.12	23.73	32.13	674.09	1.22	−4.58	−112.97
34	26.25	30.01	727.00	26.10	34.48	757.63	0.15	−4.48	−458.72
36	28.30	32.18	845.53	27.47	37.07	855.38	0.83	−4.89	−9.85
38	30.15	33.57	949.32	29.53	37.74	975.77	0.62	−4.17	−26.45

Note that the valve geometry under physiological loading conditions considerably differs from the cardioplegic valve morphology. Intraoperatively, when sizing is usually conducted, the valve is flaccid and collapsed. The actual difference in size and shape between these two states could be virtually visualized and quantified by our group for the first time [29], using novel accurate optical measurement technology. Future work includes collection of intraoperative valve shape information and the actual conducted sizing procedure to enable improved comparisons. The ultimate goal would be to translate the hitherto ambiguous intraoperative experiences of annuloplasty ring sizing to pre-repair echocardiography to enable implementation of objective planning tools with improved options for risk stratification and prediction for mitral valve repair failure.

## Conclusion

Within the scope of this paper, we showed the potential effect of different isolated sizing strategies on the mitral valve geometry. Using the height of the AML to find the optimal size of the annuloplasty ring led to the maximal reduction in mitral valve orifice area and hypothetically to the maximal increase in the coaptation area between AML and PML when compared to the other sizing methods. In contrast, the use of the AML surface area resulted in a smaller reduction in the mitral valve orifice area and a smaller increase in the coaptation area between the leaflets in theory. Sizing the mitral valve annuloplasty ring according to the intercommissural distance led to the smallest reduction in mitral valve orifice. To sum up, our results suggest that the concordance between the strategies is very limited and that they should be rather used in a combined way to make an adequate decision for the patient.

## Additional file

Additional file 1: Measurements of mitral annulus dimensions extracted from Mitralyzer software. (XLSX 42 kb)

## Abbreviations

3D TEE: Real-time three-dimensional transesophageal echocardiography
AML: Anterior mitral leaflet
CE ring: Carpentier-Edwards Physio II ring
MITK: Medical imaging interaction toolkit
PML: Posterior mitral leaflet
SAM: Systolic anterior movement
